# Adaptive Goal Processes and Underlying Motives That Sustain Mental Wellbeing and New Year Exercise Resolutions

**DOI:** 10.3390/ijerph20020901

**Published:** 2023-01-04

**Authors:** Joanne M. Dickson, Amelia Hart, Caitlin Fox-Harding, Christopher D. Huntley

**Affiliations:** 1 Psychology Division, School of Arts & Humanities, Edith Cowan University, Joondalup 6027, Australia; 2 Exercise Medicine Research Institute, Edith Cowan University, Joondalup 6027, Australia; 3 Faculty of Health and Life Sciences, School of Medicine, University of Liverpool, Liverpool L69 3GB, UK

**Keywords:** New Year exercise resolutions, goal flexibility, goal tenacity, intrinsic and extrinsic motives, mental wellbeing

## Abstract

Exercise resolutions are the most common goals people set each New Year. However, research has rarely examined adaptive goal processes and motives that may maintain wellbeing and resolution pursuit. We therefore investigated if (1) personal goal flexibility and tenacity maintain mental wellbeing and adherence to New Year exercise resolutions and if (2) underlying intrinsic and extrinsic motives maintain wellbeing and sustained exercise resolution adherence. A community sample (*N* = 297) completed an online longitudinal study. At baseline, participants listed their most important exercise-related New Year resolution. Participants then completed measures to assess goal flexibility and tenacity, resolution motives, and mental wellbeing at baseline. At three follow-up surveys over a two-month period, participants completed self-report measures of wellbeing and adherence to their exercise resolution. As predicted, goal flexibility and tenacity each independently predicted wellbeing across time. Counter to prediction, neither goal flexibility nor tenacity predicted sustained exercise adherence. Notably, underlying intrinsic motives (but not extrinsic motives) maintained mental wellbeing and exercise adherence across time. Our findings indicate that goal flexibility and tenacity are beneficial in maintaining mental wellbeing and that pursuing resolutions for internalised motives is beneficial for one’s mental wellbeing and exercise adherence.

## 1. Introduction

Personal goal striving is fundamental to the human experience. It influences everyday behaviour and promotes positive adaptions in life [1,2]. Personal goals are defined as internal representations of desired future states that involve striving toward a positive outcome [3], making them an important conduit of change in clinical and non-clinical settings. New Year resolutions represent a common form of personal goal setting in Western cultures, with over 70% of the population reported to set a resolution [4]. The popularity of New Year resolutions may be linked with the ‘fresh start effect’, where people often choose to make changes at the start of a temporal milestone, such as a Monday, or the beginning of a new calendar year [5]. New Year resolutions offer an opportunity to reflect on the past year and to consider desired outcomes people wish to pursue in the coming year [6]. As with personal goals, New Year resolutions typically require people to take goal-aligned actions and to remain resilient in overcoming difficulties or obstacles. Although people typically report a high commitment to their New Year resolutions and believe that they will achieve their resolutions, research indicates that they are not particularly good at sticking with them beyond a few weeks [6,7].

The most reported New Year resolution is exercise or physical activity [6,7,8]. Increased exercise adherence is associated with mental and physical health benefits [9]. Similar to New Year resolutions generally, and despite the benefits of exercise, people tend not to adhere to general exercise goals set throughout the year, with more than 50% reporting physical inactivity after the first month of initiating an exercise goal [10,11]. Hence, efforts to bolster exercise adherence has become a key health promotion focus [11]. A better understanding of motivational mechanisms that sustain exercise is needed to inform the ongoing development of more effective health promotion programs. New Year represents a naturally rich ecological seasonal context for setting personal goal resolutions. Whereas Dickson and colleagues’ [6] research studied New Year resolutions across a range of domains, our first aim is to investigate whether specific goal motivational mechanisms (goal flexibility, goal tenacity) predict sustained exercise resolution pursuit and mental wellbeing. Next, we aim to investigate, for the first time, whether distinct motives for pursuing exercise-related resolutions facilitate increased exercise adherence and mental wellbeing over time.

Oscarsson and colleagues found that people reporting approach-oriented resolutions (i.e., goals focused on working towards positive outcomes) across a range of domains were significantly more successful at sticking to their New Year resolutions per se, than those reporting avoidance-oriented goals (i.e., goals focused on avoiding negative outcomes) [5]. These same authors found that participants who received ‘some support’ (e.g., twelve monthly follow-ups and information about how to cope with possible obstacles when striving towards their goal) also had greater success in sticking to their resolution. Other research, without follow-up support however, found no significant associations between approach- and avoidance-oriented resolutions and sustained resolution pursuit [6]. Koestner et al. [7] investigated resolution triumph and found no significant difference between groups focused on either, ‘how they would achieve their goal’, or ‘why they wished to achieve their goal’, or ‘neither’, thus concluding that how or why people wished to achieve their goals did not appear to be relevant to resolution triumph.

Investigations of adaptive goal processes (goal flexibility, goal tenacity) have shown significant positive associations between adaptive goal processes and positive psychological outcomes, such as low depressive symptoms and high subjective wellbeing [12,13,14,15,16]. Goal flexibility refers to the ability to adjust or adapt one’s goal pursuit when confronted with obstacles, including the ability to effectively disengage from goals that are unachievable, and viewing setbacks with equanimity [14,17]. Goal tenacity on the other hand, refers to the ability to persist in striving toward achievable goals with determination under difficult conditions [17]. Goal flexibility and tenacity have each been shown to independently reduce depressive symptoms and physical ill health in older populations, and the interaction between flexibility and tenacity significantly increased these positive changes [12,15,18]. Dickson and colleagues [6] investigated goal-flexibility and tenacity in relation to New Year resolutions in a community sample. Participants’ self-generated New Year resolutions represented a range of domains (e.g., relationships, leisure, finance, work). Results found that flexible (but not tenacious) New Year resolution pursuit increased mental wellbeing across time. However, neither goal flexibility nor tenacity, or the interaction between these two goal processes, predicted sustained New Year resolution pursuit. As this was the first study to investigate New Year resolutions from a goal-motivational perspective, our first aim is to replicate this study but solely in relation to exercise resolutions.

Exercise and physical activity represent the most frequently reported New Year resolution [6]. Further, exercise is a key factor in determining mental health [19], but we know relatively little about the motivational processes that may facilitate sustained exercise engagement. In contrast to exercise, emerging research is increasingly implicating favourable and unfavourable motivational mechanisms in mental health and affective symptoms [20,21]. Although Dickson and colleagues’ [6] research found that neither goal flexibility nor tenacity predicted sustained resolution stickability across a range of resolution domains, investigations into more primary levels of motivation that energise and drive New Year resolution pursuit beyond the surface goal level warrant further investigation. Motivational systems are hierarchically organised [22]. Thus, New Year resolutions are likely to be hierarchically organised and interconnected with other levels of personal motivation. For example, an underlying motive such as, “to maintain heart health”, may motivate and drive an exercise New Year resolution such as, “to walk each day for 40 min”. Hence, our second aim is to investigate for the first time whether underlying motives for pursuing exercise resolutions predict sustained exercise adherence and mental wellbeing.

Self Determination theory (SDT) posits that people are motivated to pursue personal goals, such as New Year resolutions, for underlying intrinsic (autonomous) or extrinsic (controlled) reasons [23]. An intrinsically motivated resolution represents a goal that is pursued for personal reasons or inherent satisfaction, such as the fun, enjoyment, satisfaction, or the positive experience it provides [24], whereas an extrinsically motivated goal refers to a goal that is pursued in response to external stimuli such as, rewards, situational demands, or pressures [24]. According to SDT, human behaviour is motivated by the drive to fulfill the psychological needs for autonomy, competency, and relatedness [23]. When goals are intrinsically motivated, individuals experience interest and enjoyment, and feel more competent in what they are doing [23]. Intrinsic goal pursuit has been associated with positive psychological outcomes [23,25], and provides a deeper level of meaning [26], thus further enhancing a person’s performance when achieving their goal [24]. In contrast, extrinsic motives for goal pursuit have been associated with anxiety and depression [27,28]. Therefore, we aimed to investigate for the first time whether underlying intrinsic- and extrinsic motives maintain mental wellbeing and New Year exercise resolution adherence.

Based on motivational theory and past related empirical research, we hypothesised that goal flexibility and tenacity, measured at baseline, would each independently predict (i) increased mental wellbeing across time and (ii) sustained New Year exercise resolution stickability. We also predicted that (iii) the interaction between goal- flexibility and tenacity would further increase mental wellbeing and sustained New Year exercise resolution adherence. Finally, we hypothesised that underlying intrinsic motives (but not extrinsic motives) would independently predict (iv) increased mental wellbeing and (v) New Year resolution exercise adherence over time.

## 2. Method

### 2.1. Design

An online longitudinal survey was conducted using the Qualtrics survey platform. All participants commenced the study between the 28 December 2021 and the 31 January 2022. Baseline measures (administered at Time 1) were followed by three subsequent surveys (Time 2, 3 and 4). There was a two-week interval between Time 1 and 2, between Time 2 and Time 3, and a four-week interval between Time 3 and Time 4. The longer four-week interval between Time 3 and Time 4 was included to account for people often abandoning their New Year resolution within, or just after, the first month [29].

An a priori power calculation was conducted to determine the minimum sample required for a hierarchical regression analysis based on six predictors (including age and gender). With a desired statistical power of 0.80, a medium effect size (*f^2^* 0.15) and probability level of 0.05, at least 97 participants were required at Time 4. New Year resolution studies typically report a high attrition rate, with up to a two-thirds dropout rate [6]. Therefore, 289 participants were required at Time 1 for the present study, to allow for up to a 66% attrition rate.

### 2.2. Participants

Participants were recruited from the Australian community using advertisements on social media and organisations with a healthcare or wellness focus. The total sample comprised 297 participants, consisting of 233 females (78.5%), 62 males (20.9%), and two participants reporting as non-binary/third gender (0.7%). The proportion of males and females did not differ significantly across the four time points (*p*’s > 0.05). Participant ages ranged from 18 to 81 years (*M* = 35.22 years, *SD* = 14.21). Mean age did not differ significantly different across the four time points (*p’s* > 0.05).

### 2.3. Measures

New Year Resolution Task [6,30]. This adapted task was administered at Time 1 to elicit participants’ most important exercise-related New Year resolution. Participants were asked to, ‘think carefully about their New Year resolution(s) related to exercise, and to select the one resolution to which they are most committed.’ The exercise resolution was then recorded as a single, short written statement. Participants were asked to list a resolution that was sufficiently specific to easily assess whether they were sticking to it over the coming weeks.

New Year Exercise Resolution Intrinsic & Extrinsic Motive Ratings. Motive ratings assessed the extent to which participants pursued their listed exercise resolution for intrinsic and extrinsic reasons. Participants completed four self-report questionnaire items, informed by Sheldon and Kasser’s method of measuring goal motivation regarding people’s reasons for pursuing their goals [31,32]. Participants rated four reasons that correspond to a continuum of self-determination ranging from highly intrinsic to highly extrinsic motives, which included the following: (i) “How personally meaningful is your reason for pursuing your New Year exercise resolution?”; (ii) “To what extent are you motivated to pursue this resolution because of the personal enjoyment, satisfaction or the fun it will provide?”; (iii) “To what extent are you motivated to pursue this resolution because of the situation or someone else demands it, or because of a sense of shame, guilt or worry you’d feel if you don’t pursue it?”; (iv) “To what extent are you motivated to pursue this resolution because of some external reward you will receive (e.g., award, money)?” Each item was rated on a 7-point scale ranging from 1 (not at all) to 7 (extremely).

The two respective intrinsic items and two extrinsic items were summed to form a total intrinsic motive score, and a total extrinsic motive score. Total scores for the intrinsic and extrinsic motives each ranged from 2 to 14, with higher scores indicating greater intrinsic and extrinsic motivated exercise resolutions.

Tenacious Goal Pursuit and Flexible Goal Attainment Scale (TEN/FLEX) [17]. This measure assesses Tenacious goal pursuit (TGP: e.g., “I stick with my goals and projects, even in the face of great difficulty”) and flexible goal adjustment (FGA: e.g., “I find that even life’s troubles have their bright side”). Both TGP and FGA subscales comprise 15 items. Items are rated from 1 (‘strongly disagree’) to 5 (‘strongly agree’). TGP and FGA subscale scores ranged from 15 to 75, with higher scores reflecting greater goal tenacity and goal flexibility, respectively. TEN/FLEX subscales have acceptable reliability (all Cronbach alphas ≥ 0.80) [6,17]. The current study showed acceptable and comparable reliabilities, with Cronbach alphas, α = 0.79 for FGA and α = 0.83 for TGP.

Warwick—Edinburgh Mental Wellbeing Scale (WEMWBS) [33]. The WEMWBS assesses mental wellbeing. It consists of 14 items (e.g., “I’ve been feeling optimistic about the future”) assessing hedonic (i.e., subjective experiences of life satisfaction and happiness) and eudaimonic (i.e., mental functioning and self-realisation) perspectives of mental wellbeing [33]. Items are rated from 1 (‘none of the time’) to 5 (‘all of the time’). Total scores range from 14 to 70, with higher scores representing increased levels of mental well-being. Past research has evidenced good test-retest reliability and acceptable Cronbach alphas 0.90 or above in population samples [6,33]. The present study showed comparable and acceptable reliabilities, with Cronbach alphas ranging from α = 0.93 to α = 0.95 from Time 1 to Time 4.

Longitudinal Goal Ratings [6]. Participants rated three items applied to their New Year exercise resolution from Time 2 to Time 4 to assess resolution ‘stickability’ (Dickson et al., 2021). The items included: (1) Commitment (i.e., “To what extent are you still committed to this resolution?”), (2) Stickability (i.e., “In the last two weeks how successful have you been in sticking to this resolution?”) and (3) Effort (i.e., “In the last two weeks, to what extent have you put effort into sticking to this resolution?”). As previously noted, the final survey at Time 4 used four weeks as the timeframe. Items are rated from 1 (‘not at all’) to 7 (‘extremely’). Because of the large correlational effects found in both Dickson et al.’s [6] research (ranging from *r* = 0.58 to 0.73) and the present study (ranging from *r =* 0.47 to 0.81), the three New Year resolution goal items were summed to form a total resolution ‘stickability’ score. Cronbach’s alpha for the combined resolution ‘stickability’ variable was ≥0.90 at each time point.

An additional item was included to determine why participants had recently abandoned their exercise-related resolution (i.e., ‘If you have abandoned this resolution, please provide a brief reason why (open text box)’). Sixteen participants listed that they had abandoned their exercise resolution at Time 2, eight participants had abandoned their exercise-related resolution at Time 3, and 14 participants at Time 4. Main reasons listed for participants abandoning their exercise-related resolution were time pressures and COVID-19 difficulties.

### 2.4. Procedure

Ethical approval was granted by Edith Cowan University Human Research Ethics Committee (HREC: 2021-02874-DICKSON). The study was administered online via Qualtrics across the four time points. Participants were provided with a participation information sheet and provided consent before participating in the study. At baseline (Time 1), participants first completed demographic items, the New Year Resolution Task, followed by the exercise resolution motives, TEN/FLEX and the WEMWBS. In the follow up surveys (Time 2, Time 3 and Time 4) participants completed the Longitudinal Goal Rating items to assess resolution stickability and the WEMWBS to assess mental wellbeing.

### 2.5. Preliminary Coding and Analytic Procedures

Exploratory items were included after the New Year Resolution Task at baseline to ascertain if participants had decided on their resolution prior to the New Year: (i) “Had you decided on this exercise-related New Year resolution before beginning this survey?” and whether they had re-activated a previous resolution, and (ii) “Have you had this exercise-related New Year resolution in previous years?” Most participants had decided upon their resolution prior to the survey (*n* = 190; 63.97%), a quarter of participants (*n* = 77; 25.93%) had ‘partially’ decided on their resolution, and 29 participants (9.76%) reported that they had not decided on their exercise-related resolution prior to commencement. Approximately 60% of participants reported that they had had the same (*n* = 93; 31.31%), or partially the same (*n* = 86; 29.96%) resolution in prior years, while just over a third of participants reported that they had not the same resolution previously (*n* =118; 39.73%). Participants also rated resolution commitment, (i) “To what extent are you committed to sticking to this resolution for 2022?”, and the ‘stickability’ importance of their resolution (ii) “To what extent is sticking to this resolution important to you?” Finally, participants rated whether they had already been attempting their resolution, (iii) “To what extent have you already been trying to do this over the past year?” Participants rated their answers from 1 (‘not at all’) to 7 (‘extremely’). Mean scores indicate participants’ high commitment to the listed resolution (*M* = 5.43, *SD* = 1.13), and high importance in sticking to their resolution (*M* = 5.35, *SD* = 1.25).

Participants’ New Year resolutions were coded for (i) goal orientation (i.e., approach versus avoidance) and (ii) specificity. A resolution was coded as approach-oriented if it involved striving towards a positive outcome (e.g., ‘increase fitness and mobility’), whereas a resolution was coded as avoidance-oriented if it involved striving to prevent or inhibit a negative outcome a (e.g., ‘prevent weight gain’). Most participants listed an approach resolution (94.6%), with only 5.4% listing an avoidance resolution. A resolution was considered ‘specific’ it included a target feature or outcome with at least one of the following features: people, place, or time (e.g., ‘I will do 30-min of yoga three times a week’), whereas an exercise-related New Year resolution was coded as ‘general’ if it was described vaguely (e.g., ‘running’). Slightly more than half the participants described a ‘non-specific’ general resolution (56.6%), and the remainder described a specific resolution (43.3%). Inter-rater reliability yielded good reliability for all coding domains (all K’*s* = 1) (see Supplementary analyses Tables S1–S4).

## 3. Results

### 3.1. Data Screening

Participants were removed from the survey analysis if they did not complete the two dependent variable measures (i.e., mental wellbeing (WEMWBS) and resolution stickability). Thus, 49 participants were removed at Time 1, nine participants at Time 2, and three participants at Time 3 and Time 4. Hence, 297 participants completed Time 1, 179 (60.2%) participants completed Time 2, 134 (45.1%) participants completed Time 3, and 113 (38.0%) participants completed Time 4. There were no significant differences between those who completed all time points and non-completers on the main study variables at baseline (i.e., goal tenacity, flexible goal adjustment, resolution motives, and mental wellbeing; *p*’s > 0.05). Nor did the proportion of men and women differ significantly for completers versus non-completers (all *p’s* > 0.05). There was however a significant difference in age, with results showing participants who completed all phases of the study were younger than non-completers (*M*_age completers_ = 34.00, *SD* = 12.99 vs. *M*_age non-completers_ = 37.81, *SD* = 37.81; *t* (294) = −2.26, *p* < 0.001). Data screening showed that parametric assumptions were met for regression analysis.

Additional preliminary analyses were conducted to investigate if there were significant differences on the main study variables for those participants who had not decided on their resolution prior to beginning the survey compared to those who had decided or partially decided on their resolutions. No significant group differences were observed on goal tenacity, mental wellbeing or gender at baseline (*p*’s > 0.05). There was however a significant difference in age, goal flexibility and intrinsic motives. Participants who had decided their resolution prior to beginning the survey were significantly older (*M*_yes-decided_ = 37.61, SD = 15.20 vs. *M*_partially-decided_ = 31.90, SD = 11.48 vs. *M*_no-decided_ = *M* = 31.03, SD = 12.03, f (2,292) = 6.18, p = 0.002), reported higher goal flexibility (*M*_yes-decided_ = 50.98, SD = 6.84 vs. *M*_partially-decided_ = 48.26, SD = 7.55; f (2,293) = 4.19, p = 0.016) and intrinsic motive scores (*M*_yes-decided_ = 10.41, SD = 2.36 vs. *M*_partially-decided_ = 9.66, SD = 2.07 vs. *M*_no-decided_ = 9.24, SD = 2.17; f (2,293) = 5.30, p = 0.006) than those who had not decided or partially decided on their resolution. There were no significant differences between groups who had chosen a new resolution from those who had reactivated or partially reactivated previous resolutions (i.e., goal-flexibility, tenacity or gender, all p’s > 0.05), except for age, with participants who had the same resolution in previous years being significantly older (*M*_new-resolution_ = 37.61, SD = 15.16 vs. *M*_partially-reactivated_ = 31.90, SD = 11.48 vs. *M*_reactivated_ = 31.03, SD = 12.03; f (2,292) = 6.18, *p* = 0.002) than those who had not reactivated or partially reactivated previous resolutions.

### 3.2. Descriptive Statistics and Correlations

Descriptive statistics and Pearson’s correlations between the main study variables are presented in Table 1. Goal flexibility was significantly correlated with mental wellbeing across time, showing moderate to large effects. However, except for Time 4, flexibility was not associated with exercise resolution stickability. As expected, goal tenacity showed a significant relationship with wellbeing and stickability across time, showing small-to-medium effects. Intrinsic motives were positively correlated with wellbeing from Time 1 to Time 3 (but not Time 4), and with resolution stickability across all time points with moderate effects. Conversely, except for Time 1, extrinsic motives did not significantly correlate with either wellbeing or resolution stickability at any other time point. As expected, wellbeing ratings were significantly positively correlated across all four time points, as were stickability ratings. Wellbeing mean scores were consistent with past community samples [33]. Age significantly correlated with goal flexibility, extrinsic motives, mental wellbeing (at Time 2 and 3), and with resolution stickability at all time points. Independent *t*-tests showed no significant gender differences, except for resolution stickability at Time 3 and wellbeing at Time 2, where males reported greater levels of resolution stickability (*M*_male_ = 16.14, *SD* = 3.79 vs. *M*_female_ = 12.98, *SD =* 4.86; *t*(132) = 2.82, *p* = 0.005) and higher wellbeing *(M*_male_ = 50.48, SD = 6.88 vs. *M*_female_ = 46.63, *SD* = 46.63; *t*(175) = 2.01, *p* = 0.046) than females.

### 3.3. Do Goal Tenacity and Flexibility Predict Mental Wellbeing and Exercise Resolution Stickability?

To test our hypotheses, the first two hierarchical regressions examined if goal flexibility and goal tenacity at baseline (Time 1) independently predicted both mental wellbeing and then exercise-related New Year resolution stickability across time, while controlling for participant gender and age. We also tested whether the interaction between goal flexibility and tenacity made a significant contribution to mental wellbeing and stickability over-and-above the two independent goal variables. To aid interpretation of the interaction, variables were first mean centred, before their product was calculated. Gender and age were entered into Step 1. Goal flexibility and tenacity were entered in Step 2, and the interaction between flexibility and tenacity was entered in Step 3. A Bonferroni correction was applied to control for multiple tests at each time point. Predictor variables were considered significant if probability fell below 0.0125 (i.e., 0.05/4) when predicting mental wellbeing across the four time points, and 0.0167 (i.e., 0.05/3) for exercise resolution stickability across three time points.

The first hierarchical regression analysis tested whether goal flexibility and tenacity predicted mental wellbeing across time (see Table 2 for results). As predicted, goal flexibility significantly predicted mental wellbeing at all time points, as did goal tenacity except for Time 3. The interaction between goal flexibility and tenacity, however, did not significantly predict increased mental wellbeing at any time point.

Next, a hierarchical regression investigated whether goal flexibility and tenacity predicted exercise resolution stickability across time. As can be seen in Table 3, goal flexibility did not predict exercise resolution stickability at any time point, nor did goal tenacity, except at Time 2. The interaction between goal tenacity and goal flexibility did not predict increased exercise resolution stickability across time.

### 3.4. Do Intrinsic and Extrinsic Motives Predict Mental Wellbeing and Exercise Resolution Stickability?

Two separate multiple regression analyses were conducted to investigate if intrinsic motives (but not extrinsic motives) independently predicted increased mental wellbeing and New Year exercise resolution stickability over time, respectively. All variables were entered into the model and the same Bonferroni corrections were applied (i.e., *p* < 0.0125 for mental wellbeing and *p* < 0.0167 for ‘stickability’). The first multiple regression analysis investigated whether intrinsic (but not extrinsic motives) predicted mental wellbeing across time, while controlling for age and gender (see Table 4).

Intrinsic motives independently predicted increased mental wellbeing at Time 1 and 2 and approached significance at Time 3 and 4. Extrinsic motives negatively predicted mental wellbeing at Time 1 and Time 2 and approached significance at Time 3 and Time 4. In summary, although not significant across all time points, the results suggest a trend toward intrinsic motives being beneficial for mental wellbeing whereas extrinsic motives tend to be negatively associated with wellbeing.

The second multiple regression examined intrinsic and extrinsic motives as predictors of resolution stickability (see Table 5). As hypothesised, intrinsic motives (but not extrinsic motives) positively predicted exercise resolution stickability at all time points. Hence, the results indicate that intrinsic motives (but not extrinsic motives) are beneficial in assisting people to sustain their New Year exercise resolutions.

## 4. Discussion

Our research investigated the relationship between adaptive goal processes (goal flexibility, goal tenacity) and underlying motives (intrinsic, extrinsic) that predict mental wellbeing and New Year resolution exercise pursuit over time. As predicted, goal flexibility and goal tenacity each independently predicted mental wellbeing across time (except for one time point for goal tenacity). Counter to prediction, neither goal flexibility nor tenacity predicted exercise resolution ‘stickability’ across time (except for tenacity at Time 2). Nor did the interactive effects between goal flexibility and goal tenacity significantly predict sustained mental wellbeing or exercise resolution adherence. As hypothesized findings showed that intrinsic motives significantly predicted (and trended towards significance at later time points) increased mental wellbeing and sustained New Year resolution exercise adherence. In contrast, extrinsic motives negatively predicted mental wellbeing and did not significantly predict sustained exercise pursuit across time. In sum, our findings support the view that adaptive goal processes are key ingredients in maintaining mental wellbeing but not exercise adherence. However, underlying intrinsic motives (but not extrinsic motives) represents a key motivational determinant in sustaining both exercise resolution adherence and positive mental wellbeing over time.

### 4.1. Goal Flexibility and Tenacity as Predictors of Mental Wellbeing and New Year Exercise Resolution Stickability

The fact that goal flexibility was shown to significantly predict mental wellbeing across time is consistent with Dickson and colleagues’ [6] findings, and the view that effective goal adjustment promotes mental wellbeing [34,35,36,37]. Our findings support the view that goal flexibility, the ability to readily adapt to changing circumstances is protective in maintaining and promoting mental wellbeing [12,14,15,18]. Concordant with past research that has shown an association between goal tenacity and positive psychological outcomes in on older populations [15,18], our findings showed that goal tenacity independently predicted mental wellbeing across time. Arguably tenacious goal pursuit, itself, implies a clear goal target to strive toward, a sense of direction, industry, and purpose in life that, in turn, may maintain a sense of wellbeing. Further, exercise-related resolutions are likely to possess a clear target focus (e.g., swim every Monday morning at the local pool), which may assist and reinforce motivational persistence, relative to other resolution domains (e.g., leisure, finance, relationships). In contrast to Dickson and colleagues’ [6] research which elicited a range of resolution domains, the sole focus on exercise resolutions in the present study may account for our differing findings. The conceptual definition of goal tenacity is characterised by persistence [17], which in our study was associated with mental wellbeing. Depending on the sample population, however, it is possible that tenacity elicits rigidity rather than adaptive persistence which may explain the different findings.

Although our findings indicate that goal flexibility and tenacity are implicated in the maintenance of mental wellbeing these goal mechanisms appear to play no role in exercise adherence. These findings are in accord with Dickson and colleagues’ [6] findings which found no association between these goal mechanisms (flexibility, tenacity) and resolutions across a range of domains (e.g., finance, leisure, work, relationships). It is not clear why goal flexibility and tenacity do not facilitate exercise adherence. One possible explanation might be because individuals who adopt a flexible approach are apt to readily disengage from their exercise resolution in favour of other pursuits perceived as more important, pressing or relevant. On the other hand, tenacious goal pursuit may make it difficult to modify or effectively alter a resolution or pathway toward a resolution, even when one is failing to progress or is making poor progress. Alternatively, it is also possible that people tend to be overly optimistic and unrealistic when setting New Year resolutions, particularly during the festive holiday season, when there may be fewer competing demands and perhaps more available time.

Taken together, adaptive goal pursuit is apt to lead to increases in enjoyment, meaning, and enhanced wellbeing, even if the resolution is not successfully sustained or achieved [1,2,38]. Therefore, encouraging people to focus on adaptive goal processes, rather than outcomes, is arguably a key factor in maintaining mental wellbeing.

### 4.2. Intrinsic and Extrinsic Underlying Motives as Predictors of Mental Wellbeing and New Year Resolution Exercise Stickability

Positive associations between intrinsically motivated exercise resolutions and mental wellbeing supports the view that resolutions motivated by autonomous and intrinsic reasons such as fun, enjoyment, reward, purpose or meaning are beneficial to maintaining one’s mental wellbeing [23]. Theoretically, pursuing intrinsically motivated goal resolutions fulfils fundamental psychological needs, such as a sense of competence, which in turn promotes mental health [23,25].

Moreover, our findings indicate for the first time that intrinsic motives (but not extrinsic motives) sustain New Year exercise resolution adherence. These findings support a hierarchical view of motivation [22] and highlight the importance of examining more primary levels of motivation, that drive and energise the surface level resolution. Pursuing exercise-related New Year resolutions for intrinsically motivated reasons is apt to reinforce reward experiences and sustain motivated exercise adherence. Performing an activity for the inherent satisfaction, rather than for external motivations such as rewards or social recognition, is likely to lead to greater personal fulfilment, persistence, and enhanced engagement with a particular resolution activity [23,25,26].

In contrast to intrinsic motives, extrinsic motives were found to be detrimental to mental health over time, which is consistent with research that has shown associations between extrinsic motives and diminished mental wellbeing and increased anxiety [27,28]. Our findings indicate that striving to sustain exercise for externally driven reasons such as the situation or others demand it or for external rewards does not benefit exercise adherence.

### 4.3. Implications

Our findings have important implications for mental wellbeing, exercise behaviour practice, policy development and public health campaigns. Distinct goal motivational mechanisms, specifically, goal flexibility, goal tenacity and intrinsically motivated meaningfully resolution pursuits were each shown to maintain mental wellbeing over time. Mental health policy developments, health promotion campaigns and practitioners, would benefit from targeting and strengthening these specific goal motivational processes to promote mental health.

Benefits of sustained exercise adherence on physical health and mental wellbeing have been well documented [9]. Therefore, efforts to bolster exercise adherence has become a key health promotion focus [11]. However, research has shown that a high proportion of people typically ‘give-up’ on exercise goals, set throughout the year, within the first month [10,11]. Hence, a better understanding of specific motivational mechanisms that sustain exercise goal adherence is urgently needed. Notably, our findings indicate that pursuing exercise resolutions for intrinsically motivated reasons such as fulfilling a personal need, enjoyment or satisfaction, sustained exercise adherence and maintained mental wellbeing over time. In contrast, although extrinsic motives such as rewards or socially driven emphasis on body image or appearance may initiate setting exercise resolutions, our results found extrinsic motives do not sustain exercise adherence and are detrimental to mental health over time. Notably, these findings have important implications for informing mental health practitioners, exercise regimes, public health policy developments and public health campaigns in targeting favourable (and countering unfavourable) motivational processes to promote exercise adherence and mental health.

### 4.4. Methodological Considerations

The following limitations deserve comment. Consistent with previous New Year resolution studies, there was a relatively large attrition rate of 62% from Time 1 to Time 4. This is despite participants reporting a high level of commitment and importance to their New Year resolutions. This finding again highlights that people do not generally pursue their resolution beyond a few weeks [6,7,29]. However, it should be noted that there were no significant differences observed between those who completed all four time points and non-completers on the main study variables at baseline (i.e., goal tenacity, goal flexibility, motives and mental wellbeing). The COVID-19 pandemic may have had some impact on the study. The study was conducted during a period of relatively high infection rates in Australia [39]. Public health restrictions and both short- and long-term effects of the virus were the main reason given for abandoning a resolution, although the number of participants reporting this reason was low. Further, the key variables (i.e., goal tenacity, goal flexibility, and underlying motives) could have mitigated how participants responded to their resolutions despite the challenges of the pandemic. The present sample was predominantly female. However, there were no gender differences observed on the main study variables and gender was controlled for in the regression analyses used to test the hypotheses.

As the focus of our research was on New Year exercise resolutions pursued over a two-month period our study commenced at the beginning of the New Year. However, future research would benefit from investigating exercise goals set during other time periods such as during the winter months and for a longer duration. Given the high proportion of people who typically report recycling previous resolutions and who ‘give-up’ on their resolution pursuit, future research that examines why people fail or give up prematurely on their resolutions would further advance understanding on this topic. The present study was based on self-report measures. The inclusion of a behavioral measure(s), such as steps count per day would further strengthen the research design. It would be useful for future research to collect data on the health and clinical mental health status of participants. This would permit an investigation of potential moderating effects of these variables on the mediated relationships observed in the present study. Finally, future research that recruits a larger male sample would provide an opportunity to investigate, at a more granular level, potential gender differences in relation to distinct types of exercise and associations with motivational mechanisms, exercise adherence and mental health.

## 5. Conclusions

In sum, the study showed that while goal flexibility and tenacity maintained mental wellbeing, they were not particularly beneficial in assisting people to stick to their exercise resolution. The present study highlights the importance of investigating resolutions beyond the ‘surface’ level to examine underlying motives that may drive goal pursuit. The study distinguishes that intrinsic, but not extrinsic motives, were beneficial both for sustained exercise and wellbeing. Results indicate that to sustain exercise resolution engagement and wellbeing greater emphasis should be placed on the inherent satisfaction, personal meaning, or the benefits of exercise-related goals. The ability to maintain an exercise regimen is important in both clinical and non-clinical populations given its link to positive physical and psychological outcomes [40]. The present study highlights that setting personal resolutions or goals concordant with intrinsically meaningful motives is an important determinant in mental wellbeing and sustained exercise adherence. Future research to investigate other distinct motivational processes that may explain the relationships between maintaining exercise goals and mental wellbeing, in tandem with more objective methods (e.g., step counts), is warranted.

## Figures and Tables

**Table 1:** Descriptive Statistics and Pearson correlations between main study variables.

Variable	2.	3.	4.	5.	6.	7.	8.	9.	10.	11.	Mean (*SD*)
1. FGA	0.27 **	0.15 *	−0.16 **	0.50 **	0.45 **	0.43 **	0.48 **	0.11	0.08	0.21 *	50.09 (7.16)
2. TGP	-	0.17 **	−0.16 **	0.34 **	0.37 **	0.22 **	0.33 **	0.21 **	0.18 *	0.22 *	48.32 (7.91)
3. Intrinsic Motives		-	0.16 **	0.17 **	0.25 **	0.17 *	0.16	0.36 **	0.27 **	0.35 **	10.09 (2.31)
4. Extrinsic Motives			-	−0.18 **	−0.16 *	−0.13	−0.16	0.05	0.04	−0.03	4.11 (2.49)
5. T1 WEMWBS				-	0.77 **	0.67 **	0.69 **	0.22 **	0.19 *	0.25 **	45.91 (9.38)
6. T2 WEMWBS					-	0.72 **	0.71 **	0.22 **	0.20 *	0.31 **	47.22 (9.74)
7. T3 WEMWBS						-	0.75 **	0.18 *	0.29 **	0.32 **	47.45 (9.25)
8. T4 WEMWBS							-	0.24 *	0.28 **	0.54 **	47.25 (10.03)
9. T2 Stickability								-	0.58 **	0.48 **	13.58 (5.13)
10. T3 Stickability									-	0.58 **	13.48 (4.83)
11. T4 Stickability										-	12.49 (5.20)

Notes. * = *p* < 0.05, ** = *p* < 0.01. T = Time; FGA = Flexible Goal Adjustment; TGP = Tenacious Goal Pursuit; WEMWBS = Warwick-Edinburgh Mental Well-being Scale.

**Table 2:** Tenacious goal pursuit and flexible goal adjustment as predictors of mental wellbeing from Time 1 to Time 4.

	T1 WEMWBS (*n* = 294)					T2 WEMWBS (*n* = 176)					T3 WEMWBS (*n* = 133)					T4 WEMWBS (*n* = 112)				
Variable	*β*	*b*(*SE*)	95% CIs	*t*	*p*	*β*	*b*(*SE*)	95% CIs	*t*	*p*	*β*	*b*(*SE*)	95% CIs	*t*	*p*	*β*	*b*(*SE*)	95% CIs	*t*	*p*
Step 1	*ΔR^2^* = 0.24, *ΔF* = 3.64, *p* = 0.027					*ΔR^2^* = 0.05, *ΔF* = 4.55, *p* = 0.012					*ΔR^2^* = 0.06, *ΔF* = 4.21, *p* = 0.017					*ΔR^2^* = 0.03, *ΔF* = 1.57, *p* = 0.213				
Age	0.11	0.07 (0.04)	−0.00, 0.15	1.81	0.071	0.16	0.10 (0.05)	0.01, 0.19	2.12	0.035	0.24	0.14 (0.05)	0.04, 0.24	2.84	0.005	0.13	0.08 (0.06)	−0.04, 0.20	1.39	0.169
Gender	−0.11	−2.56 (1.33)	−5.43, −0.35	−1.93	0.054	−0.16	−3.90 (1.87)	−7.59, −0.21	−2.08	0.039	−0.03	−0.87 (2.16)	−5.14, 3.41	−0.40	0.690	−0.10	−2.53 (2.53)	−7.54, 2.49	−1.00	0.320
Step 2	*ΔR^2^* = 0.28, *ΔF* = 55.02, *p* < 0.001					*ΔR^2^* = 0.25, *ΔF* = 30.36, *p* < 0.001					*ΔR^2^* = 0.18, *ΔF* =14.68, *p* < 0.001					*ΔR^2^* = 0.27, *ΔF* = 20.11, *p* < 0.001				
Age	−0.01	−0.01 (0.03)	−0.07, 0.06	−0.27	0.788	0.09	0.06 (0.04)	−0.02, 0.14	1.38	0.169	0.20	0.12 (0.05)	0.03, 0.21	2.55	0.012	0.10	0.07 (0.05)	−0.04, 0.17	1.26	0.210
Gender	−0.08	−1.92 (1.16)	−4.20, 0.35	−1.66	0.097	−0.10	−2.43 (1.65)	−5.68, 0.83	−1.47	0.143	−0.02	−0.48 (2.01)	−4.46, 3.50	−0.24	0.812	−0.06	−1.57 (2.21)	−5.95, 2.81	−0.71	0.478
T1 FGA	0.45	0.59 (0.07)	0.46, 0.73	8.60	<0.001	0.38	0.51 (0.09)	0.34, 0.69	5.73	<0.001	0.37	0.47 (0.10)	0.26, 0.67	4.52	<0.001	0.42	0.58 (0.12)	0.34, 0.81	4.93	<0.001
T1 TGP	0.19	0.23 (0.06)	0.10, 0.35	3.64	<0.001	0.26	0.32 (0.08)	0.16, 0.49	3.85	<0.001	0.13	0.16 (0.10)	−0.03, 0.35	1.64	0.104	0.23	0.28 (0.11)	0.07, 0.50	2.66	0.009
Step 3	*ΔR^2^* = 0.00, *ΔF* = 0.02, *p* = 0.876					*ΔR^2^* = 0.01, *ΔF* = 1.98, *p* = 0.161					*ΔR^2^* = 0.00, *ΔF* = 0.00, *p* = 0.973					*ΔR^2^* = 0.00, *ΔF* = 0.22, *p* = 0.641				
Age	−0.01	−0.01 (0.03)	−0.07, 0.06	−0.26	0.794	0.10	0.06 (0.04)	−0.02, 0.14	1.48	0.142	0.20	0.12 (.047)	0.03, 0.21	2.52	0.013	0.10	0.06 (0.05)	−0.04, 0.17	1.19	0.235
Gender	−0.08	−1.93, (1.16)	−4.21, 0.35	−1.66	0.097	−0.09	−2.36 (1.64)	−5.61, 0.88	−1.44	0.153	−0.02	−0.48 (2.02)	−4.47, 3.51	−0.24	0.812	−0.06	−1.61 (2.22)	−6.00, 2.79	−0.72	0.471
T1 FGA	0.45	0.59 (0.07)	0.46, 0.73	8.50	<0.001	0.39	0.53 (0.09)	0.35, 0.70	5.86	<0.001	0.37	0.46 (0.12)	0.26, 0.67	4.41	<0.001	0.41	0.57 (0.12)	0.33, 0.80	4.74	<0.001
T1 TGP	0.19	0.23, (0.06)	0.10, 0.35	3.60	<0.001	0.27	0.34, (0.08)	0.17, 0.50	4.00	<0.001	0.13	0.16 (0.10)	−0.04, 0.35	1.61	0.109	0.22	0.28 (0.11)	0.06, 0.49	2.53	0.013
T1 FGA X T1 TGP	0.01	0.00, (0.01)	−0.01, 0.02	0.16	0.876	0.09	0.01, (0.01)	−0.01, 0.04	1.41	0.161	−0.00	0.00 (0.01)	−0.02, 0.02	−0.03	0.973	−0.04	−0.01 (0.01)	−0.03, 0.02	−0.47	0.641
Model:	*R*^2^ = 0.29, *F* (5, 288) = 23.94 ***					*R*^2^ = 0.31, *F* (5, 170) = 15.06 ***					*R*^2^ = 0.24, *F* (5, 127) = 7.85 ***					*R*^2^ = 0.29, *F* (5, 106) = 8.87 ***				

Notes. * = *p* < 0.05, ** = *p* < 0.01. *** = *p* < 0.001. T = Time; FGA = Flexible Goal Adjustment; TGP = Tenacious Goal Pursuit; WEMWBS = Warwick-Edinburgh Mental Well-Being Scale.

**Table 3:** Tenacious goal pursuit and flexible goal adjustment as predictors of exercise resolution stickability from Time 2 to Time 4.

	T2 Stickability (*n* = 176)					T3 Stickability (*n* = 133)					T4 Stickability (*n* = 112)				
Variable	*β*	*b*(*SE*)	95% CIs	*t*	*p*	*β*	*b*(*SE*)	95% CIs	*t*	*p*	*β*	*b*(*SE*)	95% CIs	*t*	*p*
Step 1	*ΔR^2^* = 0.07, *ΔF* = 6.72, *p* = 0.002					*ΔR^2^* = 0.11, *ΔF* = 8.22, *p* <.001					*ΔR^2^* = 0.07, *ΔF* = 4.00, *p* = 0.21				
Age	0.22	0.072 (0.03)	0.03, 0.12	2.98	0.003	0.24	0.07 (0.03)	0.02, 0.12	2.87	0.005	0.22	0.07 (0.03)	0.012, 0.13	2.38	0.019
Gender	−0.15	−2.03 (0.99)	−3.99, −0.08	−2.05	0.042	−0.22	−2.91 (1.10)	−5.08, −0.74	−2.66	0.009	−0.13	−1.73 (1.28)	−4.26, 0.80	−1.36	0.178
Step 2	*ΔR^2^* = 0.04, *ΔF* = 3.50, *p* = 0.032					*ΔR^2^* = 0.02, *ΔF* = 1.68, *p* = 0.190					*ΔR^2^* = 0.07, *ΔF* = 4.11, *p* = 0.019				
Age	0.21	0.07 (0.03)	0.02, 0.12	2.91	0.004	0.24	0.08 (0.03)	0.02, 0.13	2.91	0.004	0.22	0.07 (0.03)	0.012, 0.13	2.40	0.018
Gender	−0.11	−1.54 (1.00)	−3.50, 0.43	−1.54	0.125	−0.19	−2.53 (1.12)	−4.74, −0.33	−2.27	0.025	−0.10	−1.32 (1.26)	−3.82, 1.18	−1.05	0.297
T1 FGA	0.03	0.02 (0.05)	−0.09, 0.13	0.37	0.713	0.01	0.01 (0.06)	−0.10, 0.12	0.15	0.882	0.15	0.10 (0.07)	−0.03, 0.24	1.56	0.123
T1 TGP	0.19	0.13 (0.05)	0.03, 0.23	2.47	0.014	0.15	0.09 (0.05)	−0.01, 0.20	1.72	0.088	0.18	0.12 (0.06)	−0.00, 0.24	1.95	0.054
Step 3	*ΔR^2^* = 0.00, *ΔF* = 0.03, *p* = 0.873					*ΔR^2^* = 0.02, *ΔF* = 2.84, *p* = 0.095					*ΔR^2^* = 0.00, *ΔF* = 0.31, *p* = 0.579				
Age	0.22	0.07 (0.03)	0.02, 0.12	2.91	0.004	0.23	0.07 (0.03)	0.02, 0.12	2.72	0.007	0.21	0.07 (0.03)	0.01, 0.13	2.31	0.023
Gender	−0.11	−1.53 (1.00)	−3.50, 0.44	−1.53	0.127	−0.19	−2.56 (1.12)	−4.75, −0.36	−2.31	0.023	−0.10	−1.34 (1.27)	−3.85, 1.17	−1.06	0.291
T1 FGA	0.03	0.02 (0.05)	−0.09, 0.13	0.38	0.703	−0.02	−0.01 (0.06)	−0.13, 0.10	−0.19	0.850	0.14	0.097 (0.07)	−0.04, 0.23	1.43	0.157
T1 TGP	0.19	0.13 (0.05)	0.03, 0.23	2.47	0.015	0.13	0.08 (0.05)	−0.03, 0.19	1.49	0.138	0.18	0.11 (0.06)	−0.01, −0.24	1.82	0.071
T1 FGA X T1 TGP	0.01	0.00 (0.01)	−0.01, 0.01	0.16	0.873	−0.14	−0.01 (0.01)	−0.02, 0.00	−1.68	0.095	−0.05	−0.00 (0.01)	−0.02, 0.011	−0.56	0.579
Model:	*R*^2^ = 0.11, *F* (5, 170) = 4.14 **					*R*^2^ = 0.29, *F* (5, 288) = 23.94 ***					*R*^2^ = 0.10, *F* (5, 106) = 3.38 **				

Notes. * = *p* < 0.05, ** = *p* < 0.01, *** = *p* < 0.001. T = Time, FGA = Flexible Goal Pursuit, TGP = Tenacious Goal Pursuit.

**Table 4:** Intrinsic and extrinsic motives as predictors of mental wellbeing from Time 1 to Time 4.

		T1 WEMWBS (*n* = 294)					T2 WEMWBS (*n* =176)					T3 WEMWBS (*n* = 133)					T4 WEMWBS (*n* = 112)			
Variable	*β*	*b*(*SE*)	95% CIs	*t*	*p*	*β*	*b*(*SE*)	95% CIs	*t*	*p*	*β*	*b*(*SE*)	95% CIs	*t*	*p*	*β*	*b*(*SE*)	95% CIs	*t*	*p*
Age	0.06	0.04 (0.04)	−0.04, 0.11	0.97	0.335	0.12	0.08 (0.05)	−0.01, 0.17	1.71	0.090	0.21	0.13, 0.05	0.26, 0.23	2.49	0.014	0.10	0.06 (0.06)	−0.06, 0.18	1.01	0.317
Gender	−0.10	−2.27 (1.30)	−4.82, 0.29	−1.75	0.081	−0.14	−3.61 (1.82)	−7.21, −0.02	−1.98	0.049	−0.02	−0.56, 2.20	−4.90, 3.77	−0.26	0.797	−0.09	−2.36 (2.54)	−7.38, 2.67	−0.93	0.355
T1 Int Motive	0.18	0.72 (0.23)	0.26, 1.18	3.06	0.002	0.24	0.98 (0.30)	0.39, 1.56	3.29	0.001	0.16	0.66, 0.36	−0.06, 1.38	1.83	0.070	0.19	0.79 (0.43)	−0.06, 1.65	1.84	0.068
T1 Ext Motive	−0.21	−0.78 (0.22)	−1.20, −0.35	−3.58	<0.001	−0.23	−1.01 (0.32)	−1.64, −0.39	−3.20	0.002	−0.18	−0.73, 0.36	−1.45, −0.02	−2.04	0.043	−0.23	−0.97 (0.42)	−1.81, −0.14	−2.32	0.022
Model:		*R*^2^ = 0.08, *F* (4, 289) = 6.66 ***					*R*^2^ = 0.14, *F* (4, 171) = 7.03 ***					*R*^2^ = 0.10, *F* (4, 128) = 3.73 **					*R*^2^ = 0.09, *F* (4, 107) = 2.56 *			

Notes. * = *p* < 0.05, ** = *p* < 0.01. *** = *p* < 0.001. T = Time; WEMWBS = Warwick-Edinburgh Mental Well-Being Scale, Int = Intrinsic, Ext = Extrinsic.

**Table 5:** Intrinsic and extrinsic motives as predictors of resolution stickability from Time 2 to Time 4.

		T2 Stickability (*n* = 176)					T3 Stickability (*n* = 133)					T4 Stickability (*n* = 112)			
Variable	*β*	*b*(*SE*)	95% CIs	*t*	*p*	*β*	*b*(*SE*)	95% CIs	*t*	*p*	*β*	*b*(*SE*)	95% CIs	*t*	*p*
Age	0.17	0.06 (0.02)	0.01, 0.10	2.44	0.016	0.20	0.06 (0.03)	0.01, 0.11	2.40	0.018	0.15	0.05 (0.03)	−0.01, 0.11	1.64	0.104
Gender	−0.10	−1.36 (0.96)	−3.26, 0.53	−1.42	0.158	−0.18	−2.41 (1.12)	−4.62, −0.20	−2.16	0.033	−0.08	−1.04 (1.24)	−3.51, 1.42	−0.84	0.404
T1 Int Motive	0.32	0.69 (0.16)	0.38, 0.99	4.39	<0.001	0.19	0.41 (0.19)	0.04, 0.78	2.21	0.029	0.35	0.77 (0.21)	0.35, 1.19	3.65	<0.001
T1 Ext Motive	−0.02	−0.04 (0.17)	−0.37, 0.30	−0.21	0.833	−0.03	−0.06 (0.18)	−0.43, 0.30	−0.34	0.729	−0.15	−0.33 (0.21)	−0.74, 0.08	−1.59	0.115
Model:		*R*^2^ = 0.017, *F* (4, 171) = 8.57 ***					*R*^2^ = 0.15, *F* (4, 128) = 5.42 ***					*R*^2^ = 0.17, *F* (4, 107) = 5.63 ***			

Notes. * = *p* < 0.05, ** = *p* < 0.01. *** = *p* < 0.001. T = Time, Int = Intrinsic, Ext = Extrinsic.

## Data Availability

Anonymous data was collected and save on an SPSS datafile. This SPSS dataset will be deposited at Edith Cowan University’s data repository and accession numbers will be made available upon request.

## Supplementary Materials

The following supporting information can be downloaded at: https://www.mdpi.com/article/10.3390/ijerph20020901/s1, Table S1: Commitment, importance, and previous attempts as predictors of mental wellbeing at Times 1–4; Table S2: Commitment, importance, and previous attempts as predictors of New Year resolution stickability at Times 2–4; Table S3: Resolution orientation (approach vs. avoidance), resolution specificity (specific vs. general) as predictors of mental wellbeing at Times 1–4; Table S4: Resolution orientation (approach vs. avoidance), resolution specificity (specific vs. general) as predictors of stickability at Times 2–4.
