# Supplementary material for: Adaptive Goal Processes and Underlying Motives That Sustain Mental Wellbeing and New Year Exercise Resolutions
Source: Int J Environ Res Public Health. 2023 Jan 4;20(2):901. doi: 10.3390/ijerph20020901 (PMC9858624; doi:10.3390/ijerph20020901)
Supplement: Supplementary file 1 [file ijerph-20-00901-s001.zip › ijerph-2099942-supplementary.pdf]

Adaptive goal processes and underlying motives that sustain mental wellbeing and New Year exercise resolutions

Supplementary Analyses.

The following tables are included within this document:

1. **Table S1**. *Commitment, importance, and previous attempts as predictors of mental wellbeing at Times 1-4.*
2. **Table S2**. *Commitment, importance, and previous attempts as predictors of New Year resolution stickability at Times 2-4.*
3. **Table S3.** *Resolution orientation (approach vs. avoidance), resolution specificity (specific vs. general) as predictors of mental wellbeing at Times 1-4.*
4. **Table S4.** *Resolution orientation (approach vs. avoidance), resolution specificity (specific vs. general) as predictors of stickability at Times 2-4.*

Table S1. *Commitment, importance, and previous attempts as predictors of mental wellbeing at Times 1-4.*

	**T1 WEMWBS (*n* = 296)**					**T2 WEMWBS (*n* =178)**					**T3 WEMWBS (*n* = 133)**					**T4 WEMWBS (*n* = 114)**				
**Variable**	***β***	***b(SE)***	**95% CIs**	***t***	***p***	***β***	***b(SE)***	**95% CIs**	***t***	***p***	***β***	***b(SE)***	**95% CIs**	***t***	***p***	***β***	***b(SE)***	**95% CIs**	***t***	***p***
Age	0.086	0.09 (0.04)	-0.02, 0.13	1.45	0.147	0.16	0.10 (0.05)	0.00, 0.20	2.01	0.046	0.20	0.12 (0.05)	0.01, 0.23	2.24	0.027	0.15	0.10 (0.06)	-0.03, 0.22	1.49	0.139
Gender	-0.10	-0.10 (1.33)	-4.87, 0.36	-1.70	0.090	-0.15	-3.77 (1.89)	-7.49, -0.04	-1.99	0.048	-0.03	-0.70 (2.20)	-5.04, 3.70	-0.32	0.752	-0.10	-2.63 (2.59)	-7.77, 2.51	-1.01	0.313
Commitment	0.12	0.12 (0.66)	-0.35, 2.26	1.43	0153	0.07	0.61 (0.97)	-1.31, 2.52	0.63	0533	-0.07	-0.65 (1.04)	-2.72, 1.41	-0.63	0531	0.09	0.89 (1.27)	-1.63, 3.40	0.70	0.486
Importance	-0.07	-0.07 (0.59)	-1.68, 0.67	-0.85	0.395	0.02	0.11 (0.79)	-1.44, 1.67	0.15	0.885	0.13	0.98 (0.82)	-0.65, 2.60	1.19	0.236	-0.12	-0.91 (1.01)	-2.91, 1.08	-0.91	0.366
Attempt	0.09	0.09 (0.36)	-0.17, 1.24	1.49	0.137	-0.05	-0.28 (0.50)	-1.28, 0.71	-0.56	0.576	0.06	0.33 (0.58)	-0.81, 1.47	0.57	0568	-0.02	-0.14 (0.69)	-1.51, 1.22	-0.21	0.835
Model:		R^2 = 0.44, *F* (5,288) = 2.65*					R^2 = 0.24, *F* (5,170) = 1.99					R^2 = 0.07, *F* (5,127) = 2.04					R^2 = 0.04, *F* (5,106) = 0.79			

Notes.

* = $p < 0.05$, ** = $p < 0.01$. T = Time, WEMWBS = Warwick-Edinburgh Mental Wellbeing Scale. Bold and underlined = significant at $p = 0.05 / 4 = 0.0125$ (i.e., Bonferroni correction applied for multiple tests).

Table S2. *Commitment, importance, and previous attempts as predictors of New Year resolution stickability at Times 2-4.*

	**T2 Stickability (*n* = 178)**					**T3 Stickability (*n* = 134)**					**T4 Stickability (*n* = 114)**				
**Variable**	***β***	***b(SE)***	**95% CIs**	***t***	***p***	***β***	***b(SE)***	**95% CIs**	***t***	***p***	***β***	***b(SE)***	**95% CIs**	***t***	***p***
Age	0.14	0.05, 0.02	0.00, 0.09	2.04	0.043	0.17	0.05 (0.03)	-0.00, 0.10	1.95	0.054	0.14	0.05 (0.03)	-0.02, 0.11	1.49	0.140
Gender	-0.12	-1.59, 0.88	-3.33, 0.14	-1.81	0.072	-0.18	-2.40 (1.07)	-4.53, - 0.28	-2.24	0.027	-0.08	-1.10 (1.26)	-3.60, 1.41	-0.87	0.387
Commitment	0.35	1.65, 0.45	0.76, 2.54	3.65	< 0.001	0.14	0.66 (0.51)	-0.35, 1.67	1.30	0.195	0.12	0.61 (0.62)	-0.61, 1.84	0.99	0.325
Importance	0.04	0.14, 0.37	-0.58, 0.87	0.39	0.697	0.08	0.30 (0.40)	-0.49, 1.10	0.76	0.451	0.02	0.10 (0.49)	-0.88, 1.07	0.20	0.846
Attempt	0.17	0.52, 0.23	0.06, 0.99	2.24	0.027	0.13	0.40 (0.28)	-0.16, 0.95	1.41	0.162	0.19	0.63 (0.34)	-0.04, 1.29	1.87	0.064
Model:	R^2 = 0.54, *F* (5,170) = 13.70***					R^2 = 0.19, *F* (5,127) = 5.79 ***					R^2 = 0.14, *F* (5,106) = 3.43**				

Notes.

* = $p < 0.05$, ** = $p < 0.01$. *** = $p < 0.001$. T = Time. Bold and underlined = significant at $p = 0.05 / 3 = 0.0167$ (i.e., Bonferroni correction applied for multiple tests).

Table S3. *Resolution orientation (approach vs. avoidance), resolution specificity (specific vs. general) as predictors of mental wellbeing at Times 1-4.*

	**T1 WEMWBS (*n* = 294)**					**T2 WEMWBS (*n* =176)**					**T3 WEMWBS (*n* = 133)**					**T4 WEMWBS (*n* = 112)**				
**Variable**	β	***b(SE)***	**95% CIs**	***t***	***p***	β	***b(SE)***	**95% CIs**	***t***	***p***	β	***b(SE)***	**95% CIs**	***t***	***p***	β	***b(SE)***	**95% CIs**	***t***	***p***
Age	0.11	0.07 (0.04)	-0.01, 0.14	1.84	0.067	0.18	0.11 (0.05)	0.02, 0.20	2.37	0.019	0.26	0.15 (0.05)	0.05, 0.26	2.91	0.004	0.17	0.11 (0.06)	-0.02, 0.23	1.73	0.086
Gender	-0.12	-2.72 (1.35)	-5.36, -0.67	-2.02	0.045	-0.13	-3.39 (1.94)	-7.21, 0.43	-1.75	0.082	-0.01	-0.23 (2.33)	-4.84, 4.38	-0.10	0.922	-0.06	-1.50 (2.67)	-6.79, 3.79	-0.56	0.575
Resolution Orientation	-0.10	-4.05 (2.45)	-8.88, 0.79	-1.65	0.101	0.01	0.56 (3.83)	-7.01, 8.13	0.15	0.884	0.05	2.60 (4.49)	-6.28, 11.49	0.58	0.563	0.05	2.86 (5.41)	-7.86, 13.58	0.53	0.598
Resolution Specificity	-0.09	-1.67 (1.12)	-3.87, 0.53	-1.49	0.137	-0.17	-3.36 (1.45)	-6.21, -0.50	-2.32	0.021	-0.05	-0.87 (1.66)	-4.15, 2.40	-0.53	0.598	-0.14	-2.81 (1.98)	-6.73, 1.11	-1.42	0.158
Model:	R^2 = 0.04, *F* (4,289) = 2.87*					R^2 = 0.08, *F* (4,171) = 3.75**					R^2 = 0.07, *F* (4,128) = 2.26					R^2 = 0.05, *F* (4,107) = 1.42				

Notes.

* = $p < 0.05$, ** = $p < 0.01$, *** = $p < 0.001$. T = Time, WEMWBS = Warwick-Edinburgh Mental Wellbeing Scale. Bold = significant at $p = 0.05 / 4 = 0.0125$ (i.e., Bonferroni correction applied for multiple tests).

Table S4. *Resolution orientation (approach vs. avoidance), resolution specificity (specific vs. general) as predictors of stickability at Times 2-4.*

	**T2 Resolution Stickability (*n* = 176)**					**T3 Resolution Stickability (*n* = 133)**					**T4 Resolution Stickability (*n* = 112)**				
**Variable**	***β***	***b(SE)***	**95% CIs**	***t***	***p***	***β***	***b(SE)***	**95% CIs**	***t***	***p***	***β***	***b(SE)***	**95% CIs**	***t***	***p***
Age	0.22	0.07 (0.02)	0.03, 0.12	3.02	0.003	0.24	0.08, 0.03	0.02, 0.13	2.85	0.005	0.28	0.09 (0.03)	0.03, 0.15	3.02	0.003
Gender	-0.18	-2.41 (1.03)	-4.34, -0.38	-2.34	0.021	-0.19	-2.46, 1.18	-4.79, -0.13	-2.09	0.039	-0.07	-0.95 (1.32)	-3.56, 1.66	-0.72	0.472
Resolution Orientation	-0.13	-3.54 (2.04)	-7.76, 0.48	-1.74	0.084	0.10	2.46, 2.27	-2.04, 6.95	1.08	0.281	0.06	1.67, 2.67	-3.63, 6.96	0.62	0.534
Resolution Specificity	-0.12	-1.20 (0.77)	-2.72, 0.31	-1.57	0.119	0.00	0.01, 0.84	-1.64, 1.67	0.02	0.987	-0.24	-2.50, 0.98	-4.44, -0.56	-2.56	0.012
Model:	R^2 = 0.10, F (4, 171) = 4.58**					R^2 = 0.12, F (4, 128) = 4.38**					R^2 = 0.13, F (4, 107) = 3.96**				

Notes.

* = $p < 0.05$, ** = $p < 0.01$. *** = $p < 0.001$. T = Time. Bold = significant at $p = 0.05 / 3 = 0.0167$ (i.e., Bonferroni correction applied for multiple tests).
